# Targeted Prostate Health Checks for prostate cancer in men at high risk: a feasibility pilot in primary and secondary care in North East London

**DOI:** 10.3399/BJGPO.2025.0219

**Published:** 2026-06-17

**Authors:** Muhammad Haider, Apple Dumagat, James SA Green, Paula Allchorne, Angela S Wong, Asad Ashraf, Gopal Mehta, Anil Shah, Oluwabunmi Olajide, Rhian Gabe, Fiona M Walter, Yin Zhou, Stafford Scott, Sara Paparini, Benjamin W Lamb, Prabhakar Rajan, for the North East London Cancer Alliance Targeted Prostate Health Check Group

**Affiliations:** 1 Centre for Cancer Cell and Molecular Biology, Barts Cancer Institute, Queen Mary University of London, London, UK; 2 Department of Urology, Barts Health NHS Trust, London, UK; 3 Department of Urology, Homerton Healthcare NHS Foundation Trust, London, UK; 4 North East London Cancer Alliance, NHS North East London, London, UK; 5 Department of Gastroenterology, Barts Health NHS Trust, London, UK; 6 E4 Primary Care Network, London, UK; 7 London Fields Primary Care Network, London, UK; 8 NW2 Primary Care Network, London, UK; 9 Havering Crest Primary Care Network, London, UK; 10 Centre for Evaluation and Methods, Wolfson Institute of Population Health, Queen Mary University of London, London, UK; 11 Centre for Cancer Screening, Prevention and Early Diagnosis, Wolfson Institute of Population Health, Queen Mary University of London, London, UK; 12 Tottenham Rights, London, UK; 13 SHARE Collaborative, Centre for Public Health and Policy, Wolfson Institute of Population Health, Queen Mary University of London, London, UK; 14 Division of Surgery and Interventional Science, University College London, London, UK; 15 Department of Urology, University College London Hospitals NHS Foundation Trust, London, UK

**Keywords:** prostate cancer, prostate-specific antigen, ethnicity, deprivation, primary healthcare, general practitioners, targeted screening, health check

## Abstract

**Background:**

Prostate-specific antigen (PSA)-based prostate cancer (PCa) screening risks overdiagnosis and overtreatment. PCa disproportionately affects Black men, those with a family history (FH) of the disease, and *BRCA1/2* gene variant carriers. Risk-adapted approaches are gaining interest but are underexplored.

**Aim:**

To assess the feasibility of PSA-based Targeted Prostate Health Checks (TPHCs) for men at high PCa risk, compare invitation methods, and assess sociodemographic variations.

**Design & setting:**

Prospective feasibility cohort study in four primary care networks (PCNs) in North East London.

**Method:**

Men aged 45–69 years from Black ethnic group, or with a positive PCa FH, were identified via primary care records and invited by the PCN to one of two TPHCs: (i) telephone-first (phone consultation followed by hospital-based PSA testing); or (ii) test-first (community-based PSA testing followed by phone consultation). Elevated PSA prompted multiparametric magnetic resonance imaging (mpMRI), and prostate biopsy if malignancy was suspected.

**Results:**

Of 2400 invitees, 398 (16.6%) attended. Attendance was higher with the test-first than telephone-first TPHC (22.9% versus 11.2%, *P*<0.001). Only 51.4% of participants met eligibility criteria owing to inaccurate FH coding, although men who did not meet the eligibility criteria were offered PSA tests. Black men had lower prior PSA testing (55.7% versus 82.5%) and higher deprivation than White men. Elevated PSA occurred in 6.0% of participants (*n* = 22), with five PCa diagnoses (1.4%).

**Conclusion:**

Identification of men at high PCa risk is feasible using age and ethnicity primary care data, but FH coding is unreliable. Test-first invitations improved engagement. Disparities affecting Black men highlight the need for tailored outreach, and better coding of risk factors will facilitate risk-adapted screening.

## How this fits in

PSA-based prostate cancer screening risks overdiagnosis and overtreatment. Black men and those with a positive family history are at higher risk. This UK study shows PSA-based Targeted Prostate Health Checks using age and ethnicity primary care data are feasible, with a ‘test-first’ approach improving uptake. Unreliable family history coding and persistent inequalities highlight the need for better outreach and data recording.

## Introduction

Prostate cancer (PCa) is the most common cancer in men and the second leading cause of male cancer deaths in the UK.^1^ Major risk factors are increasing age,^2^ being from a Black ethnic group,^3^ germline *BRCA1/2* gene mutations,^4^ and a family history (FH) of PCa.^5^ Early stage PCa is curable, and most PCa-related deaths occur from metastatic disease.^6^ Large randomised trials of prostate-specific antigen (PSA)-based screening show conflicting mortality outcomes and highlight risks of overdiagnosis and overtreatment.^7–9^ The UK National Screening Committee (NSC) does not recommend population-wide PSA screening, but recently issued a recommendation for targeted screening for *BRCA*2 gene mutation carriers with a family history of breast, ovarian, pancreatic, or PCa.^10^

There are currently conflicting UK guidelines on opportunistic PSA testing. The National Institute for Health and Care Excellence (NICE) suggests PSA testing only for men with urinary or advanced PCa symptoms, using age-specific referral thresholds.^11^ Asymptomatic men aged >50 years can also request a PSA test.^12^ The NHS Prostate Cancer Risk Management Programme (PCRMP) provides primary care professionals with guidance on counselling of asymptomatic men aged ≥50 years who ask about PSA testing for PCa^13^ and recommends a 3 ng/ml threshold for secondary care referral, also supported by the NHS England Clinical Expert Group (CEG).^14^ In contrast, European Association of Urology (EAU) guidelines support early PSA testing in well-informed men at elevated PCa risk based on age, ethnicity, FH, and genetic risk factors.^6^

Black men (of Black Caribbean and Black African heritage) have higher rates of PCa incidence,^15^ metastasis,^16^ and mortality compared with White men.^3,15^ These disparities are associated with socioeconomic factors, such as access to health care and deprivation. Increasing deprivation is linked to lower PCa incidence but higher mortality.^17^ This may reflect lower levels of PSA testing^18^ and reduced access to radical treatment for high-risk or locally advanced disease among Black men and those from more deprived areas.^19^ Other contributors include genetic risk, variation in PCa phenotypes,^20^ lower screening participation, limited PCa awareness, aversion to digital rectal examination (DRE), and distrust from prior healthcare discrimination.^21^

As such, some advocate for individualised early detection guidelines for Black men and risk-adapted screening.^22,23^ A recent Prostate Cancer UK (PCUK) consensus paper recommended proactive primary care discussions with Black men about PCa risk and PSA testing.^24^ Black men remain underrepresented in screening trials, with only 4% participation in the Prostate, Lung, Colorectal, and Ovarian (PLCO) Cancer Screening trial, and no ethnicity data in the European Randomized study of Screening for Prostate Cancer (ERSPC) or the Cluster Randomized Trial of PSA Testing for Prostate Cancer (CAP).^7–9^

Carriers of pathogenic *BRCA2* gene variants have an approximately 8.6-fold increased risk in men aged ≤65 years^4^ and a 20% lifetime risk of PCa.^25^ An international, multicentre cohort study (IMPACT), demonstrated that *BRCA2* variant carriers have higher PCa incidence, are diagnosed younger, and are more likely to have clinically significant PCa (csPCa) than non-carriers.^26^ PCa has a strong hereditary component, with twin studies suggesting genetic factors account for around 57% of risk variation.^27^

North East London (NEL) is a large, diverse, and economically deprived area, with a population over 2 million, of which over 50% are of a minority ethnic group, and containing four of the sixth most deprived boroughs in London.^28^ Consistent with existing literature,^3,15,29,30^ we previously identified healthcare inequalities within Urgent Suspected Cancer (USC) prostate pathway referrals in NEL: Black men had the highest age-adjusted PCa incidence rates compared with White men (around two times higher) and Asian men (around three times higher), were diagnosed at younger ages, and came from the most deprived backgrounds.^31^ To address these inequalities, PCa early detection outreach strategies for minority ethnic communities have been piloted by others across the UK and include ‘drop-in’ clinics,^32^ community-based clinics,^33,34^ and a mobile ‘Man Van’.^35^

The aim of this study was to assess the feasibility of two PSA-based Targeted Prostate Health Check (TPHC) approaches that used primary care records to identify men at high PCa risk across four NEL primary care networks (PCNs), and to compare attendance patterns and sociodemographic variation.

## Method

### Recruitment

Between February 2024 and September 2024, individuals at high PCa risk, as defined by EAU guidelines,^36^ were identified from primary care records across NEL by PCNs. Inclusion criteria were men aged 45–69 years, either from a Black (Black British, Black African, and Black Caribbean) ethnic group or any ethnic group with a positive FH of PCa amongst first-degree male relatives. . Exclusion criteria were known PCa diagnosis, PSA <3 ng/ml in the past 12 months (this cut-off was chosen based on a previous similar TPHC showing a 19% PCa prevalence in men with a PSA between NICE age-specific PSA but ≥3 ng/ml),^33^ ongoing treatment for incurable malignancy, and severe frailty or limited life expectancy (based on clinician assessment). Only three men across participating PCNs were coded as carrying *BRCA1/2* variants, and none met the other high risk inclusion criteria, so were excluded in this feasibility pilot.

Following discussions with patient and public involvement and engagement (PPIE) partners consisting of Black men (led by SP and public co-author SS), two TPHC recruitment approaches were implemented: (i) participants from London Fields, Newham North West 2 (NW2), and Havering Crest PCNs were invited by text, followed by letters and phone calls to non-responders, for a secondary care telephone assessment, PSA test at a hospital-based phlebotomy service, and a follow-up telephone clinic (‘telephone-first’); or (ii) participants from E4 PCN were invited by text for a PSA test at a local primary care phlebotomy service, followed by a secondary care telephone consultation (‘test-first’). Pre-test counselling was delivered either during the telephone clinic (telephone-first) or via a website link with an online video featuring Black men (test-first) (see Supplementary Material 2). Accessibility services differed between models, with translated website versions and text-to-speech available in the test-first model, and a telephone interpreter available in the telephone-first model. In the telephone-first model, exclusions occurred before PSA testing; in the test-first model, exclusions were applied retrospectively. Men with miscoded FH but within the eligible age range (50–69 years) were offered PSA testing. Those with elevated NICE-defined age-specific PSA levels (Appendix 1) were referred through existing USC pathways for multiparametric magnetic resonance imaging (mpMRI) and transperineal prostate biopsy (if indicated), in line with standard guidelines.^37^

### Data collection

Data collected from hospital records for TPHC attendees included age, postcode, any PSA testing history, PSA level, mpMRI, and biopsy results. Self-reported FH of PCa, self-defined ethnic group (primary care ethnicity data were not available), and International Prostate Symptom Score (IPSS)^38^ were also recorded. Ethnic subgroups (for example, Black African or Black Caribbean) were grouped under broader categories (for example, Black); those from other ethnic backgrounds were categorised as ‘any other ethnicity’ owing to low numbers. Deprivation level at the Lower-layer Super Output Area (LSOA), based on home postcode, was determined using the 2019 Index of Multiple Deprivation (IMD), a composite score measuring seven domains (income, employment, education, health, crime, housing and services, and living environment).^39^ Sociodemographic data were not available for individuals at high risk identified from primary care who did not attend a TPHC.

### Analysis

Statistical analysis was performed using StataNow (version 18.5), with *P*<0.05 indicating statistical significance. The one-sample Kolmogorov–Smirnov test assessed normality of continuous data. Age, ethnic group distributions, PSA uptake, and previous PSA testing rates were compared between TPHCs using the χ^2^ test. The Wilcoxon test was used to compare IPSS scores and number of previous PSA tests between groups. Fisher’s exact test was used to compare deprivation quintiles across groups. Tables were created using Microsoft Excel (version 2412). All data were stored on secure hospital servers.

## Results

The number of eligible men identified through the telephone-first and test-first approaches was 1294 and 1106, respectively. Of 2400 invitees, 437 men (18.2%) consented by either requesting a telephone appointment in the telephone-first TPHC or attending for PSA testing in the test-first TPHC (Figure 1). To evaluate the effectiveness of each approach, we compared attendance rates between TPHCs. In the telephone-first approach, 184 men requested an appointment, however, 39 did not attend, resulting in 145 attendances (11.2%). In the test-first approach, 253 men underwent PSA testing, and all (22.9%) attended subsequent appointments. In total, 398 (16.6%) men attended an appointment across both approaches; attendance was significantly higher in the test-first than the telephone-first TPHC (*P*<0.001). Of the 398 attendees, a total of 30 (7.5%) were subsequently excluded from downstream analyses (Figure 1). In the telephone-first TPHC eight men were excluded owing to clinical criteria and eight by patient choice. In the test-first TPHC, 14 men were excluded owing to clinical criteria. In this feasibility pilot, non-Black men who did not meet the eligibility criteria owing to incorrectly coded FH data but who were within the eligible age range were not excluded.

**Figure 1.**
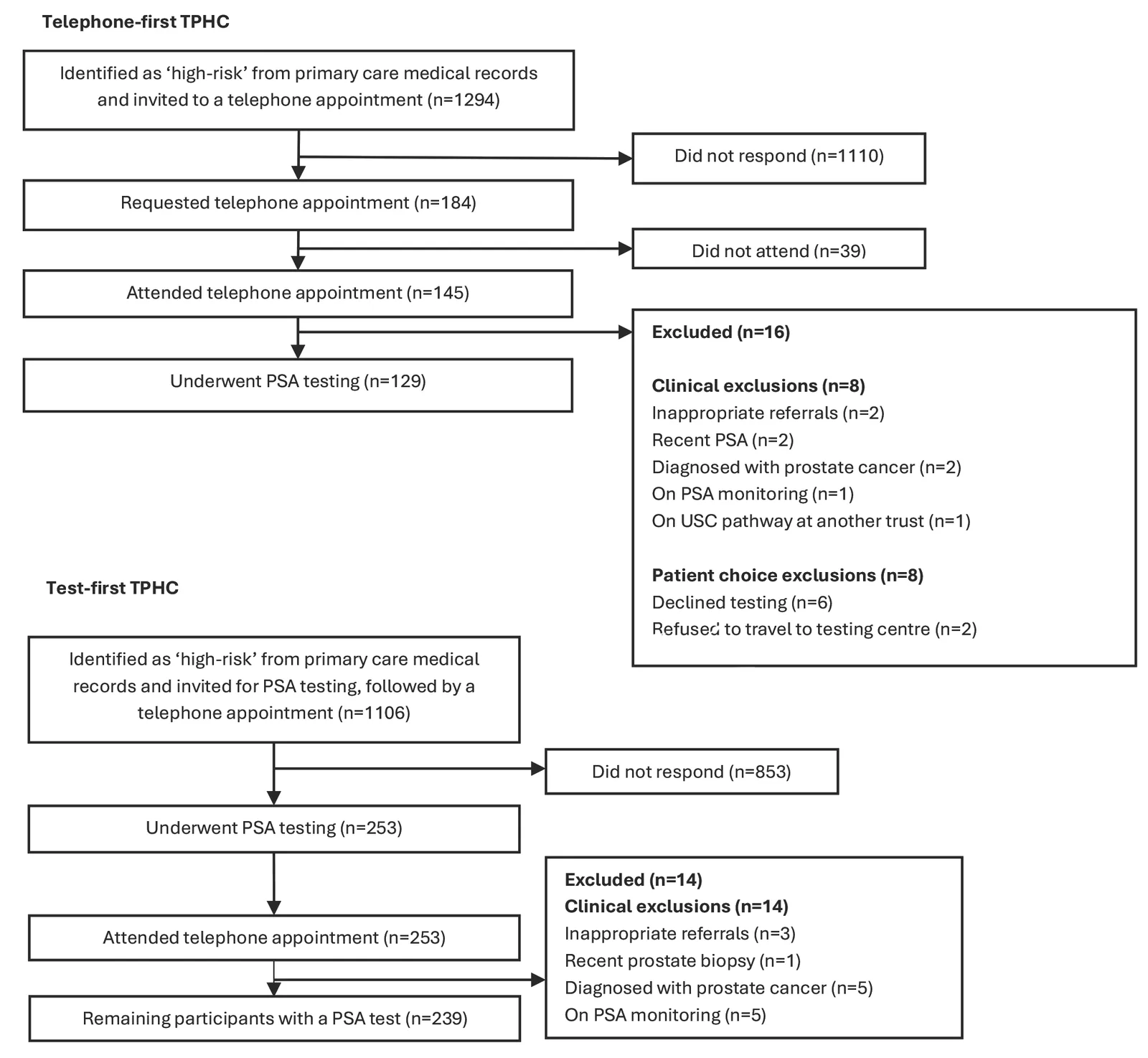
Adapted CONSORT flow diagram comparing telephone-first and test-first Targeted Prostate Health Check pathways.^52^

Baseline demographics of the final 368 participants are shown in Table 1. The majority (189, 51.4%) of participants were aged 55–64 years, with a median age of 59 years (interquartile range [IQR] 55-64.8p). Men from White ethnic groups made up the largest proportion (*n* = 177, 48.1%), followed by men from Black ethnic groups (*n* = 149, 40.5%). Of men from non-Black ethnic groups, only 40 (18.3%) self-reported a FH of PCa among first-degree male relatives. Consequently, only 189 (51.4%) of 368 of participants actually met the initial ‘at high risk’ eligibility criterion.

**Table 1.** Results of the Targeted Prostate Health Check (TPHC) by invitation method

	All	Results by invitation method		
	Total	Telephone-first approach	Test-first approach	*P* value
Number of patients (percentage of total cohort)	368 (100%)	129 (35.1%)	239 (64.9%)	-
**Age, years**				
45–54	87 (23.6%)	49 (38.0%)	38 (15.9%)	
55–64	189 (51.4%)	62 (48.1%)	127 (53.1%)	
≥65	92 (25.0%)	18 (14.0%)	74 (31.0%)	
Median (IQR)	59 (55–64.8)	56 (51–61)	61 (57–65)	<0.001
				
**Ethnic group**				
White	177 (48.1%)	16 (12.4%)	161 (67.4%)	
Black	149 (40.5%)	111 (86.0%)	38 (15.9%)	
Any other ethnicity	42 (11.4%)	2 (1.6%)	40 (16.7%)	<0.001
				
**Proportion of participants in each IMD deprivation quintile**				<0.001
1^st^ (most deprived)	35 (9.5%)	27 (20.9%)	8 (3.3%)	
2^nd^	199 (54.1%)	79 (61.2%)	120 (50.2%)	
3^rd^	103 (28.0%)	21 (16.3%)	82 (34.3%)	
4^th^	27 (7.3%)	1 (0.8%)	26 (10.9%)	
5^th^ (least deprived)	1 (0.3%)	0 (0%)	1 (0.4%)	
Missing	3 (0.8%)	1 (0.8%)	2 (0.8%)	
				
**Median IPSS score (IQR)**	0 (0–3)	1.5 (0–7)	0 (0–1)	<0.001
**Proportion of participants with any previous PSA test**	72.0%, *n* = 265	43.4%, *n* = 56	87.4%, *n* = 209	<0.001
**Median number of previous PSA tests (IQR)**	1 (0–2)	0 (0–1)	2 (1–3)	<0.001
**Proportion of non-Black participants with positive family history (FH) of PCa**	18.3%, *n* = 40	44.4%, *n* = 8	15.9%, *n* = 32	<0.001
**Median PSA (ng/ml) (IQR)**	1.1 (0.7–2.0)	1.1 (0.6–2.0)	1.1 (0.6–2.0)	N/A
**Number of participants with elevated PSA (ng/ml)**	22 (6.0%)	11 (8.5%)	11 (4.6%)	N/A
>2.5 (40–49 years)	0	0	0	
>3.5 (50–59 years)	9	5	4	
>4.5 (60–69 years)	11	5	6	
>6.5 (70–79 years)	2	1	1	
				
**Number of participants who underwent mpMRI**	18 (4.9%)	9 (7.0%)	9 (3.8%)	N/A
**Number of participants who underwent biopsy**	9 (2.4%)	2 (1.6%)	7 (2.9%)	N/A
**Number of participants diagnosed with PCa**	5 (1.4%)	1 (0.8%)	4 (1.7%)	N/A
**Gleason Grade Group (GG)1**	2	1	1	
≥GG2	3	0	3	

N/A = not applicable; statistical testing was not performed owing to small sample size. IMD = Index of Multiple Deprivation. IPSS = International Prostate Symptom Score. mpMRI = multiparametric magnetic resonance imaging. PCa = prostate cancer. PSA = prostate-specific antigen

There was a statistically significant difference in the ethnic groups of participants across the two approaches (*P*<0.001). In the telephone-first TPHC, the majority (86.0%) were Black men, whereas in the test-first TPHC, the majority (67.4%) were White men. In total, 82.2% of participants in the telephone-first TPHC were in the top two most deprived quintiles, compared with 53.6% in the test-first TPHC.

Compared with men of any other ethnicity or White men, Black men were younger (median = 57 years versus 59.5 years versus 62 years; *P*<0.001) and from more deprived backgrounds (*P*<0.001, see supplementary material 3); 79.2% of Black men were in the top two most deprived quintiles compared with 53.7% and 50.0% of White men and men from any other ethnicity, respectively. The proportion of Black men who had undergone a previous PSA test was significantly lower (55.7%) compared with White men (82.5%) and men of any other ethnicity (85.7%) (*P*<0.001).

Baseline demographics of participants by risk group are shown in Table 2. Compared with men at low risk (who did not meet these criteria), men at high risk (who met the initial eligibility criteria) were younger (median age 58 years [IQR 52-62] versus 62 years [IQR 57-66], *P*<0.001), had lower prior PSA testing rates (60.8% versus 83.8%, *P*<0.001) and were from more deprived backgrounds (*P*<0.001), 74.1% of high-risk men were in the top two most deprived quintiles compared with 52.5% of men at low risk.

**Table 2.** Results of the Targeted Prostate Health Check (TPHC) by risk group

	All	Results by risk group		
**Characteristic**	**Total**	**High (Black men or those with a PCa FH)**	**Low risk (all others)**	***P*** **value**
Number of patients (percentage of total cohort)	368 (100%)	189 (51.4%)	179 (48.6%)	
**Age, years**				
45–54	87 (23.6%)	62 (32.8%)	25 (14.0%)	
55–64	189 (51.4)	96 (50.8%)	93 (52.0%)	
≥65	92 (25.0%)	31 (16.4%)	61 (34.1%)	
Median (IQR)	59 (55–64.8)	58 (52–62)	62 (57–66)	<0.001
**Ethnic group**				
White	177 (48.1%)	33 (17.5%)	144 (80.4%)	
Black	149 (40.5%)	149 (78.8%)	0 (0%)	
Any other ethnicity	42 (11.4%)	7 (3.7%)	35 (19.6%)	<0.001
**Proportion of participants in each IMD deprivation quintile**				<0.001
1^st^ (most deprived)	35 (9.5%)	29 (15.3%)	6 (3.4%)	
2^nd^	199 (54.1%)	111 (58.7%)	88 (49.2%)	
3^rd^	103 (28.0%)	41 (21.7%)	62 (34.6%)	
4^th^	27 (7.3%)	6 (3.2%)	21 (11.7%)	
5^th^ (least deprived)	1 (0.3%)	0 (0%)	1 (0.6%)	
Missing	3 (0.8%)	2 (1.1%)	1 (0.6%)	
**Median IPSS score (IQR)**	0 (0–3)	0 (0–5)	0 (0–1)	0.0033
**Proportion of patients with previous PSA test**	72.0%, *n* = 265	60.8%, *n* = 115	83.8%, *n* = 150	<0.001
**Mean number of previous PSA tests (SD)**	1.71 (1.97)	-	-	-
**Proportion of patients with positive family history (FH) of PCa**	17.4%, *n* = 64	-	-	-
**Median PSA (IQR)**	1.1 (0.7–2.0)	1.1 (0.6–2.1)	1.1 (0.7–-2.0)	0.9457
**Number of participants with elevated PSA (ng/ml)**	6.0%, *n* = 22	14 (7.4%)	8 (4.5%)	0.2347
>2.5 (40–49 years)	0	0	0	
>3.5 (50–59 years)	9	8	1	
>4.5 (60–69 years)	11	5	6	
>6.5 (70–79 years)	2	1	1	
**Number of participants who underwent mpMRI**	18 (4.9%)	13 (6.9%)	5 (2.8%)	0.0694
**Number of men who underwent biopsy**	9 (2.4%)	6 (3.2%)	3 (1.7%)	0.3522
**Number of patients diagnosed with PCa**	5 (1.4%)	3 (1.6%)	2 (1.1%)	0.6971
**Gleason Grade Group (GG)1**	2	1	1	
≥GG2	3	2	1	

FH = family history. IMD = Index of Multiple Deprivation. IPSS = International Prostate Symptom Score. mpMRI = multiparametric magnetic resonance imaging. PCa = prostate cancer. PSA = prostate-specific antigen

Of the 368 participants, 6.0% (*n* = 22) had a raised PSA, 4.9% (*n* = 18) underwent mpMRI, and 1.4% (*n* = 5) were diagnosed with PCa. Of the five PCa diagnoses, two were Gleason Grade Group (GG) 1 and 3 had ≥GG2. Two of the PCa diagnoses were in Black men (one with a positive FH), while the remaining three were in non-Black men (two with negative FH).

## Discussion

### Summary

We demonstrate the feasibility of two PSA-based TPHCs using primary care records to identify men at high PCa risk, compare attendance, and assess sociodemographic variation. Compared with the telephone-first TPHC, the test-first TPHC achieved higher attendance (22.9% versus 11.2%). This may reflect the simplicity of single-step, community-based PSA testing compared with the multi-step telephone-first TPHC, which likely led to greater attrition. A positive FH of PCa in first-degree male relatives appears to be unreliably coded (18.3% accuracy), and *BRCA*1/*2* status is largely absent.

The telephone-first TPHC included a higher proportion of Black men, while the test-first TPHC included a higher proportion of White men. White men were far more likely to have had a previous PSA test and their higher proportion in the test-first TPHC likely explains the higher proportion of prior PSA tests in the test-first TPHC. Higher rates of past testing may also have contributed to the greater uptake observed in the test-first TPHC, as men who have been tested before may be more willing to be tested again. Participation may also have been influenced by socioeconomic factors, as men in the test-first TPHC came from less deprived areas, which may reflect greater PCa awareness. However, as the two TPHCs were conducted in different PCNs and baseline sociodemographic data (for example, ethnic group, IMD) for invitees was unavailable, the reasons for these differences remain unclear.

Among TPHC attendees, Black men were from the most deprived backgrounds, and had lower rates and fewer instances of prior PSA testing than White men. While younger age of participants may partly explain this, Black men are less likely to engage in PCa screening than White men, regardless of age.^40^ These disparities may also help explain why men who met the initial high-risk eligibility criterion were younger, more deprived, and, despite being at higher risk, less likely to have had a previous PSA test as the higher-risk group included 78.8% Black participants, whereas the lower-risk group was 80.4% White.

### Comparison with existing literature

Previously published PSA-based TPHC feasibility pilots (Table 3) used various engagement strategies including the following: a nurse-led community ‘Man Van’;^35^ nurse-led ‘drop-in’ PCa risk assessments;^32^ and community-based TPHCs coordinated by a third-party virtual healthcare provider.^41^ Of the three, only the community-based clinics reported an uptake rate (33%). In the UK-based CAP trial, men aged 50–69 years were identified via GP practices, and 40% responded after a single invitation to attend a nurse-led clinic for PSA testing.^42^ However, responders were from less deprived areas than non-responders.^43^ Therefore, the lower response rate in our TPHC may also reflect high social deprivation in NEL,^44^ which is associated with lower PSA testing rates in the UK.^18,45^

**Table 3.** Comparison with other published Targeted Prostate Health Check (TPHC) projects

Study	Eligibility criteria	Ethnic group profile of sample	PSA cut-off for investigation	Response rate	Total PSA tests, *n*	Men with elevated PSA, *n* (% of total tests)	MRIs, *n* (% of total PSA tests)	Clinically significant cancers diagnosed, *n* (% of total PSA tests)
McAllister *et al* ^32^	BME males aged between 45 years and 75 years, however screened anyone who attended	Not provided	NICE-age specific cut-offs	Not provided	312	14 (4.5%)	Not reported	5 (1.6%)
Langley *et al* ^41^	Men aged 50–70 years, or 45–70 years if Black or positive family history	Black (4.7%), Other (95.3%)	NICE-age specific cut-offs and ≥3 ng/ml	33%	18 317	678 (3.7%) above NICE threshold (referred straight on USC pathway) and 187 (1.0%) ≥3 ng/ml	817 (4.5%)	211 (1.2%)
Moghul *et al* ^35^	Black men aged >45 years and any other ethnicity aged >50 years	White 53%, Black 30%, Other 17%	NICE-age specific cut-offs	(Not applicable: patients self-registered via paper forms or email)	420	56 (13.3%)	48 (11.4%)	10 (2.4%)
Haider *et al*	Black men or men of any ethnicity with positive family history aged 45–69 years, however screened anyone who attended	White 48%, Black 41%, Other 11%	NICE-age specific cut-offs	16.6%	368	22 (6.0%)	18 (4.9%)	3 (0.8%)

BME = Black and Minority Ethnic. MRI = magnetic resonance imaging. NICE = National Institute for Health and Care Excellence. PSA = prostate-specific antigen. USC = urgent suspected cancer

To engage underserved communities, a ‘Man Van’ clinic was deployed in targeted areas,^35^ with Black men comprising 33% of attendees, nearly double their proportion in local GP populations (17.1%). A community-based TPHC in the ethnically and socioeconomically diverse London Borough of Newham used multiple advertising channels and achieved 46% attendance from Black African or Black Caribbean men.^46^ Advertising accounted for 47% of referrals but produced only short-lived increases, while 24% attended through word of mouth, highlighting the importance of peer encouragement.

Our findings align with prior research showing unreliable primary care recording of PCa risk factors. FH of PCa is poorly recoded, with one study reporting missing FH data in 98.4% of early onset PCa cases,^47^ another study found FH recording in only 0.27% of >400 000 opportunistic UK PSA tests.^48^ Previous UK-based TPHC projects^33,35^ (Table 3) did not report FH coding accuracy. *BRCA1/2* status was not recorded, likely owing to the fact that UK testing is typically limited to those with a pre-test probability above 10%,^49^ and most eligibility criteria apply to women, who account for 69% of *BRCA* tests.^50^ A study of 25 million UK primary care records found ethnicity data missing in 21.8% of patients,^51^ and we may have therefore failed to invite more Black men owing to inaccurate coding of ethnic group. Hence, relying entirely on primary care records for FH, ethnic group, and *BRCA*1*/2* mutation status in any future risk-adapted PCa screening programme may continue to contribute to healthcare inequalities.

### Strengths and limitations

To our knowledge, this is the first UK study to explore the feasibility of PSA-based TPHCs using primary care records to identify men at high PCa risk. However, there are several limitations: carriers of pathogenic *BRCA1/2* mutations were excluded, and FH appeared unreliably coded. Ethnic group data accuracy could not be guaranteed and may have led to eligible men being missed. A broad ethnic classifier (‘any other ethnicity’) was used, grouping together diverse populations that have different risk profiles. The two TPHCs were conducted in different PCNs with significant differences in sociodemographics, which limits the conclusions that can be drawn from direct comparison of invitation methods. Sociodemographic data on non-attenders was unavailable, limiting interpretation of non-participation and demographic differences between attenders and non-attenders.

### Implications for research and practice

Electronic identification of men at high risk of PCa is feasible using age and ethnic group primary care data, but FH and *BRCA1/2* status are unreliably coded. Therefore, targeted PCa screening for carriers of *BRCA2* in line with NSC recommendations will be challenging to implement at a primary care level. The overall TPHC attendance rate was low (16.6%), which may be explained by socioeconomic deprivation within an ethnically-diverse NEL region. Direct PSA test-first invitations with local phlebotomy could lead to improved attendance. Black men had the lowest rates of PSA testing and higher socioeconomic deprivation highlighting the need for tailored outreach. Further research is needed to optimise engagement strategies for PCa screening in diverse, high-risk populations, alongside improved primary care coding of risk factors.

## Data

The dataset relied on in this article is available from the corresponding author on reasonable request.
